# Effect of Debittering with Different Solvents and Ultrasound on Carotenoids, Tocopherols, and Phenolics of *Lupinus albus* Seeds

**DOI:** 10.3390/antiox11122481

**Published:** 2022-12-16

**Authors:** Lorenzo Estivi, Davide Fusi, Andrea Brandolini, Alyssa Hidalgo

**Affiliations:** 1 Department of Food, Environmental and Nutritional Sciences (DeFENS), Università degli Studi di Milano, Via Celoria 2, 20133 Milan, Italy; 2 Research Centre for Animal Production and Aquaculture (CREA-ZA), Council for Agricultural Research and Economics, Via Piacenza 29, 26900 Lodi, Italy

**Keywords:** citric acid, flavonoids, free phenolics, insoluble-bound phenolics, lupin, sodium chloride, soluble-conjugated phenolics, sonication

## Abstract

Lupin seeds represent a rich nutritional source of bioactive compounds, including antioxidant molecules such as carotenoids, tocopherols, and phenolics. However, before consumption, the lupin seeds must be debittered in order to remove their bitter and toxic alkaloids. This study analyzed the impact on the bioactive compounds of *Lupinus albus* seeds of a recent time- and water-saving debittering method, which employs alternative washing solutions (0.5% or 1% of either NaCl or citric acid), with or without the assistance of ultrasound. The results were compared with those of two control methods using water or a NaCl solution. The sonication, when it was significant, led to a large loss of bioactive compounds, which was most likely due to its extraction capability. The seeds that were debittered without ultrasound presented high concentrations of tocopherols (172.8–241.3 mg/kg DM), carotenoids (10.9–25.1 mg/kg DM), and soluble-free (106.9–361.1 mg/kg DM), soluble-conjugated (93.9–118.9 mg/kg DM), and insoluble-bound (59.2–156.7 mg/kg DM) phenolics. The soluble-free fraction showed the greatest loss after a prolonged treatment. Overall, debittering with citric acid or NaCl preserved the highest concentration of antioxidant compounds by shortening the treatment time, thus preventing extensive leaching.

## 1. Introduction

Lupin seeds are eaten as snacks, as salads, or as garnishes, and their flour is often used in the manufacturing of sauces, drinks, cheese, meat, tofu, pasta, cakes, biscuits, tortillas, muffins, and bread, etc., [1,2,3,4,5,6]. The seeds are characterized by high contents of proteins, lipids, and other bioactive compounds [7]. The proteins are suitable for the preparation of gluten-free foods for coeliac persons [8], and the protein isolates have desirable physical, technological, and functional properties, such as foaming, gelling, emulsifying, and water absorptive capacities [7,9,10]. Additionally, lupin protein hydrolysates showed promising health properties as anxiolytic [11], as nutraceutical able to prevent the early stages of atherosclerosis [12], as functional ingredients in foods for diabetic people [13], etc.

Tocopherols, carotenoids, and phenolic compounds are among the bioactive compounds that are present in lupin seeds [14,15,16]. Tocopherols and carotenoids are lipid-soluble, while phenolics (i.e., phenolic acids and flavonoids [17]) are water-soluble molecules; however, all of them have antioxidant activity and exert many positive effects on human health [18].

The major hindrance to human consumption is the presence, in most varieties, of bitter and often toxic quinolizidine alkaloids, which are synthesized by the plant as protection against pathogens and pests. Several technological processes, mainly relying on repeated washings with water, are employed in order to remove the alkaloids and render the seeds suitable for eating. Unfortunately, the few existing low-alkaloid varieties [19,20] are more susceptible to pests and diseases, are less productive, and tend to revert to bitterness [21]. Low-frequency ultrasound (US), which is a technology that is employed in order to improve compound extraction [22,23], has been tried with discordant results in lupin seed debittering [24,25].

Debittering modifies the seeds’ composition because some water-soluble molecules (e.g., minerals, sugars, and oligosaccharides) are washed away; accordingly, their removal leads to an increase in lipids and proteins for a concentration effect [26,27,28]. Debittering also affects the bioactive composition of the seeds, as it can remove or degrade part of the antioxidant compounds [29,30] and reduce the nutritional value of lupin-based foods, hence the necessity to develop debittering processes that are able to shorten the treatment time, reduce the water consumption, and minimize the bioactive compound loss.

A recent study proposed a more efficient debittering procedure [25], using different washing solutions (0.5% and 1% NaCl or citric acid) with or without ultrasound assistance. The sonication did not accelerate debittering, but the 1% citric acid solution saved 88 h and 65 L of water per kg of dry lupin compared to the water control method [28,29], and 13 h and 31 L of water per kg of dry lupin compared to the salt solution control method [31]. The aim of this work was to study the effects of the proposed debittering processes and the control methods with water or salt on the content of the antioxidant bioactive compounds (carotenoids, tocopherols, and phenolics) of *Lupinus albus* seeds.

## 2. Materials and Methods

### 2.1. Materials

Two different lots (Lot 1 and Lot 2) of *Lupinus albus* seeds from Chile, which were obtained from Colombo Legumi e Frutta Secca (Modica, Italy), were analyzed.

### 2.2. Methods

#### 2.2.1. Debittering

The seeds were debittered in batches of about 100 g following three different approaches as follows:

- Control method with water, according to Erbaş [28], with minor modifications [27], as follows: the seeds were hydrated (1:6 *w*/*v* seeds:water ratio) for 12 h at room temperature, then cooked in boiling water (hydrated seeds:water 1:3 *w*/*v*) for 1 h, replacing the water after 30 min, and rinsed with water (cooked seeds:water 1:3 *w*/*v*) for 5 days at room temperature, changing the water every 12 h.
- Control method with 0.5% sodium chloride solution, as described by Villacrés et al. [31], as follows: The seeds were hydrated for 8 h at 80 °C (1:3 *w*/*v* seeds:0.5% NaCl solution ratio), then the liquid was changed and the seeds were cooked for 1 h at 91 °C (1:3 *w*/*v*), renewing the solvent after 30 min. Five washes were then carried out using 0.5% NaCl solution at 35 °C for up to 28 h (first and second wash: 1:15 *w*/*v*, 3 h each; third wash: 1:5 *w*/*v*, 16 h; fourth wash: 1:7.5 *w*/*v*, 3 h; fifth wash: 1:5 *w*/*v*, 3 h). Two final washes with water at 18 °C (seeds:water 1:5 *w*/*v*), the first lasting 18 h and the second 3 h, were carried out to eliminate the excess salinity.
- Experimental method. A detailed description and a flowsheet of the proposed debittering approach is shown in Estivi et al. [25]. The following two different solutions were employed: 1% NaCl and 1% citric acid. Additionally, two different treatments were applied, without and with ultrasound, utilizing a UP400St ultrasonic homogenizer (Hielscher Ultrasonics GmbH, Teltow, Germany) operating at 24 kHz and mounting a 14 mm diameter probe to treat the seeds during washing (60% amplitude) and cooking (100% amplitude). After the last washing, 0 to 2 soakings with distilled water were carried out for 12 h to remove salt or citric acid from the seeds. For Lot 1, the final washing started at 25.5 h to interrupt the process after 28.5 h or 45 h. For Lot 2, the seeds were sampled at the end of the first (t_1_ = 45 h) and second (t_2_ = 57 h) final soaking.

Two trials were performed to assess the following:

(1) The influence of sonication (with or without) and solvent (1% NaCl or 1% citric acid) on antioxidants. The seeds of Lot 1 were debittered following the experimental method proposed by Estivi et al. [25]. To achieve an identical and low residual alkaloid content, a soaking time of 45 h was applied for 1% NaCl and of 28.5 h for 1% citric acid solution. The control was debittered with water, according to Erbaş [28], with minor modifications [27].
(2) The effect of solvent (1% NaCl or 1% citric acid) and total soaking time (45 or 57 h) on antioxidant content. The seeds of Lot 2 were debittered by the experimental method [25], but without sonication. These soaking times achieved very low residual alkaloid contents for the citric acid solution and for the NaCl solution, respectively [25]. The seeds that were debittered by the control method with water [27,28] and by the reference method with sodium chloride solution [31] provided the controls.

The seeds of Lot 1 were dried for 8 h at 60 °C in a Venticell 55 ventilated oven (MMM-Group, Planegg/München, Germany), while the seeds of Lot 2 were lyophilized in an Edwards 304 freeze dryer (Atlas Copco, Stockholm, Sweden). The dried seeds were ground with an MDI 204 disc mill (Buhler, Uzvil, Switzerland) and stored at −20 °C until analysis.

#### 2.2.2. Moisture

The moisture was determined gravimetrically according to the official method AOAC 925.10 [32].

#### 2.2.3. Carotenoids and Tocopherols

The carotenoids and tocopherols were extracted as outlined by Panfili et al. [33]. All tests were carried out in duplicate. Briefly, 1 g of sample was saponified under nitrogen for 45 min at 70 °C with 2.5 mL of ethanolic pyrogallol (60 g/L), 1 mL of ethanol (95%), 1 mL of sodium chloride (10 g/L), and 1 mL of potassium hydroxide (600 g/L). After cooling in an ice bath, 7.5 mL of sodium chloride (10 g/L) was added. The suspension was extracted twice with 15 mL hexane:ethyl acetate (9:1 *v*/*v*). The organic layer was collected and evaporated under vacuum and nitrogen drying. The residue was dissolved in 2 mL hexane:isopropyl alcohol (90:10 *v*/*v* for carotenoids, 99:1 *v*/*v* for tocopherols) and filtered through a 0.22 μm PTFE membrane.

Carotenoid quantification was performed by NP-HPLC as described in Brandolini et al. [34], using the following: an Adamas^®^ Silica column 250 × 4.6 mm, 5 μm, and guard cartridge 10 × 4.6 mm, 5 μm (Sepachrom SRL, Rho, Italy); a column oven at 20 °C L-2300 Elite LaChrom (Hitachi, Tokyo, Japan); mobile phase, hexane:isopropyl alcohol (95:5 *v*/*v*); flow rate, 1.50 mL/min; pump L-2130 Elite LaChrom (Hitachi, Tokyo, Japan). The carotenoids were detected at 445 nm with a system including the following: injector Rheodyne with a 50 µL loop; pump L-2130 Elite LaChrom (Hitachi, Tokyo, Japan); PDA 2996 Detector (Waters Chromatography Division, Millipore, Milford, UK); the HPLC system was controlled by the software Empower 2 (Waters Chromatography Division, Millipore, Milford, UK). For peak quantification, β-carotene (0.15–1.5 mg/L; Sigma, St. Louis, MO, USA), β-cryptoxanthin (0.02 and 0.13 mg/L; Extrasynthese, Genay, France), lutein (0.30–3.00 mg/L; Fluka, St. Louis, MO, USA), and zeaxanthin (0.05–1.03 mg/L; Extrasynthese, Genay, France) calibration curves were built. The total carotenoids were computed as the sum of the different compounds.

Tocopherol quantification was performed by NP-HPLC, as performed by Varas Condori et al. [35], using the following: an Adamas^®^ Silica column 250 × 4.6 mm, 5 μm, and guard cartridge 10 × 4.6 mm, 5 μm (Sepachrom SRL, Rho, Italy); mobile phase, hexane:ethyl acetate:acetic acid (97.3:1.8:0.9, *v*/*v*/*v*); flow rate, 1.60 mL/min; pump L-2130 Elite LaChrom (Hitachi, Tokyo, Japan); injector Rheodyne with a 50 µL loop; fluorimetric detector Jasco 821 FP Intelligent Spectrofluorometer (Jasco, Tokyo, Japan), at excitation-emission wavelengths of 290 nm and 330 nm, respectively, controlled by the software Empower 2 (Waters Chromatography Division, Millipore, Milford, UK) through the SAT/IN (Waters Chromatography Division, Millipore, Milford, UK) interface. As a reference, α-tocopherol (0.40–110 mg/L; Fluka BioChemika, Buchs, Switzerland), β-tocopherol (0.38–72.2 mg/L; Supelco, Bellefonte, PA, USA), γ-tocopherol (0.20–23.2 mg/L; Supelco, Bellefonte, PA, USA), and δ-tocopherol (0.05–9.35 mg/L; Supelco, Bellefonte, PA, USA) standard curves were constructed. Total tocopherols were computed as the sum of the different homologues. The results are reported as mg/kg dry matter (DM).

#### 2.2.4. Phenolic Compounds

The analysis of the soluble-free, soluble-conjugated, and insoluble phenolic compounds was performed by reverse-phase HPLC. The phenolics were obtained as follows: exactly 1 g of sample was extracted three times with 15 mL of 80% methanol. After centrifugation at 11,200× *g*, the pooled supernatants (which contained the soluble-free or the soluble-conjugated fractions) and the sediment (which contained the insoluble-bound fraction) were recovered. For the free phenolic extraction [36] the supernatant was evaporated under vacuum at 35 °C for 50 min with a Laborota 4000 rotary evaporator (Heidolph, Milan, Italy), dried under nitrogen flux, resuspended in 2 mL of methanol:water (8:2 *v*/*v*), and filtered with a 0.45 μm PTFE membrane. For the soluble-conjugated phenolics [37], the supernatant was evaporated under vacuum at 35 °C for 18 min. Then, the samples were digested with 15 mL of 4 M NaOH under nitrogen for 4 h at room temperature and were continuously shaken, were brought to pH 1.5–2 with 6 M HCl, and extracted twice with 20 mL of diethyl ether:ethyl acetate (1:1 *v*/*v*). The extracts were clarified with sodium sulphate, filtered through 110 μm glass fiber (Whatman, Maidstone, England), evaporated under vacuum at 35 °C for 5 min, resuspended in 2 mL of methanol:water (1:1 *v*/*v*), and filtered with a 0.22 μm PTFE membrane (Millipore, Carrigtwohill Co., Cork, Ireland). For the analysis of the insoluble-bound phenolic compounds [36], the sediment was digested and then extracted as outlined above for the soluble-conjugated phenolics.

The HPLC analysis [37] was performed using a column SepaChrom C18, 250 mm × 4.6 mm, 5 µm (Sepachrom SRL, Italia) and a precolumn SepaChrom C18, 10 mm × 4.6 mm, 5 µm (Sepachrom SRL, Italy) thermostated at 30 °C with a column oven L-2300 Elite LaChrom (Hitachi, Tokyo, Japan). The system included the following: injector Rheodyne with a 20 µL loop; pump L-2130 Elite LaChrom (VWR, Hitachi, Tokyo, Japan); Diode Array Detector L-2450 Elite LaChrom (Merck, Hitachi, Tokyo, Japan); and software EZChrom Client/Server versione 3.1.7). Gradient elution was achieved using acetonitrile (A) and 1% (*v*/*v*) formic acid in water (B) mobile phases at 1 mL/min flow rate, following the gradient profile of 0–10 min from 10% to 25% A, 10–20 min linear rise to 60% A, and 20–30 min linear rise to 70% A, followed by 10 min reverse to 10% A, with 5 min of equilibration time. The identity of the compounds was verified by the congruence of retention times and UV/VIS spectra with those of 33 pure authentic standards. The unidentified peaks were pooled according to their absorbance spectrum and their similarity to the standard spectra, designating them as the respective standard, but with the addition of the term “derivative”, as proposed by several authors [14,38,39,40]. The calibration curves of the identified phenolics were constructed using standards (Sigma-Aldrich, St. Louis, MO, USA) recorded at 280 nm for catechin (13.9–99.2 mg/L), genistein (27.5–110 mg/L), and naringenin (2.25–9.00 mg/L), at 320 nm for apigenin (1.00–20.0 mg/L) and sinapic acid (0.50–21.4 mg/L), and at 360 nm for diosmin (5.24–104.8 mg/L). The results are expressed as mg/kg DM.

### 2.3. Statistical Analysis

To compare the samples within each test, after verifying their normal distribution, the data underwent one-way analysis of variance (ANOVA), while to evaluate the effect of treatment (T) and solvent (S), the data were processed by two-way ANOVA. When significant differences (*p* ≤ 0.05) were detected, Fisher’s LSD test at 95% significance level was applied. All analyses were performed with the Statgraphics Centurion XVI statistical program (Statgraphics Technologies Inc., The Plains, VA, USA). The means, standard deviations, and standard errors were computed using Excel^®^ software (version 2016, Microsoft, Redmond, WA, USA).

## 3. Results and Discussion

### 3.1. Effect of Sonication and Solvents on Antioxidant Compounds

#### 3.1.1. Carotenoids and Tocopherols

Table 1 shows the carotenoid and tocopherol content of the Lot 1 seeds that were debittered with solutions of 1% NaCl for 45 h or 1% citric acid for 28.5 h, either without or with sonication, and with the water control method for comparison. The durations of the treatments were chosen based on the minimum time that was required to reach the same level of residual alkaloids (about 1 g alkaloids/kg DM), according to Estivi et al. [25].

The total carotenoid content varied from 10.9 to 13.2 mg/kg DM, which were values that were superior to those (1.9–5.3 mg/kg) that were recorded by Briceño Berru et al. [15] in water-debittered seeds of *Lupinus albus*. In the literature, scarce information on carotenoid content exists, and often refers to bitter seeds. The most abundant carotenoid in our treated seeds was lutein (6.8–8.7 mg/kg DM; Table 1), which was also reported for bitter and debittered *L. albus* samples by Briceño Berru et al. [15], for bitter seeds of *L. luteus* by Fernández-Marín et al. [41], and of *L. mutabilis* by Wang et al. [42]. However, other authors [42,43] found that zeaxanthin was the main carotenoid in bitter seeds of *L. albus*, *L. luteus*, *L. angustifolius*, and *L. mutabilis*.

The two-way ANOVA (Supplementary Table S1) of the lupins that were debittered with the experimental method showed that both the treatment and the solvent influenced the total carotenoid content, while their interaction was not significant. Sonication led to values that were inferior to those of the non-sonicated seeds, but the differences were minimal. The one-way ANOVA (not shown) and the LSD test (Table 1) evidenced that the seeds that were debittered with 1% citric acid and US showed the lowest content of all of the identified carotenoids, bar β-cryptoxanthin; the other treatments preserved the total carotenoids slightly better.

Only tocopherols were recovered from the debittered *L. albus* seeds, which was observed by Boschin and Arnoldi [44], Fernández-Marín et al. [41], and Briceño Berru et al. [15]; no tocotrienol was identified. Total tocopherol contents were 195.4 mg/kg DM after the control treatment and 172.8–192.0 mg/kg DM after the experimental treatments. These concentrations were in the range (135.9–222.7 mg/kg DM) of the debittered samples that were analyzed by Briceño Berru et al. [15] and were superior to those of the bitter *L. albus* seeds (107.0–153.0 mg/kg DM) that were reported by Annicchiarico et al. [45]. The most abundant compound was γ-tocopherol, which represented over 99% of the total tocopherols. Its concentration (169.0–188.7 mg/kg DM) was lower than that (201–516 mg/kg DM) reported by Martínez-Villaluenga et al. [46] for bitter *L. albus* seeds, but was similar to that (131.0–218.4 mg/kg DM) detected in debittered seeds by Briceño Berru et al. [15], and superior to the results (61.2–130.0 and 73.6 mg/kg) that were reported for bitter seeds of *L. albus* by Boschin and Arnoldi [44] and by Lampart-Szczapa et al. [47], respectively.

The two-way ANOVA (Supplementary Table S1) did not show significant effects of the treatment, the solvent, or their interaction on tocopherol content, except for α-tocopherol (interaction) and β-tocopherol (treatment and solvent). In fact, β-tocopherol was slightly more abundant after debittering without the use of US, and with the citric acid solution, compared to the sodium chloride solution.

The detrimental effect of ultrasound on carotenoids in the presence of citric acid could be hastily attributed to the production of radicals from the homolytic cleavage of water [48,49]. Carotenoids are degraded by reactive oxygen species undergoing unspecific chain-breaking events [50]; however, the frequency that we employed (24 kHz) results in minimal development of hydroxyl radicals [48,51]. Most likely, citric acid intensified the ultrasound probe erosion [52], releasing metal ions, which catalyze the formation of superoxide anion [53]. In addition, carotenoids quench singlet oxygen with reaction rates two orders of magnitude higher than α-tocopherol [53]; this may explain why the decrease was significant for carotenoids and not for tocopherols.

Most of the literature deals with non-debittered *L. albus* seeds; nevertheless, the debittered samples that we studied had similar or, in some cases, higher tocopherol and carotenoid contents. Apparently, debittering did not lead to a drastic loss of lipophilic antioxidants, which are insoluble in aqueous solutions and, therefore, are not washed away. On the contrary, the leaching of alkaloids and other water-soluble compounds may concentrate tocopherols and carotenoids, as observed by Brandolini et al. [14] and Briceño Berru et al. [15].

#### 3.1.2. Phenolic Compounds

Table 2 reports the free, the conjugated, and the bound phenolic compounds content of the Lot 1 seeds after debittering with solutions of 1% NaCl for 45 h or of 1% citric acid for 28.5 h, either without or with sonication. Most of the peaks that were detected in the chromatograms did not correspond to the retention times and spectra of the standards, apart from genistein. Therefore, they were recorded as derivatives of sinapic acid and the flavonoids genistein, naringenin, diosmin, and apigenin, as well as of catechin. Apigenin derivatives were found by Karamać et al. [54] and Siger et al. [55] in different lupin species, and by Czubinski et al. [16] in *L. mutabilis*, while Kalogeropoulos et al. [56] identified small amounts of sinapic acid (0.4 mg/kg DM), catechin (1.9 mg/kg DM), and genistein (1.6 mg/kg DM) in *L. albus*.

The overall total polyphenols of the samples that were debittered with the experimental method varied between 434.8 and 539.3 mg/kg DM; the soluble-free fraction was the most abundant and represented 54.2–67.0% of the total, while the soluble-conjugated and the insoluble-bound fractions were 18.5–23.0% and 11.0–22.8%, respectively. For *L. albus* species, the only literature data available deals with non-hydrolyzed methanol extracts, which are akin to the soluble-free fraction, generally in non-debittered seeds. The samples that were debittered with the experimental method showed lower quantities of free polyphenols (235.7–361.1 mg/kg DM) than those (418.5–446.7 mg/kg DM and 537.4 mg/kg DM) that were reported for non-debittered *L. albus* seeds by Siger et al. [55] and Kalogeropoulos et al. [56], respectively. The soluble-conjugated and insoluble bound phenolic compounds contents, instead, were 85.3–118.9 mg/kg DM and 59.2–101.2 mg/kg DM, respectively.

The two-way ANOVA (Supplementary Table S2) showed that, for the free phenolic compounds, the effects of the treatment (T), the solvent (S), and their interaction (T × S) were significant (*p* ≤ 0.05), except for the genistein derivatives (T not significant), the sinapic acid derivatives, and the total free phenols (T × S not significant). The LSD test indicated that the ultrasound treatment, when it was significant, determined a lower free phenols content, which is attributable to its enhanced extraction capacity [23,57]. The 1% citric acid and the 1% NaCl solutions led to average residual soluble-free phenolic contents of 343.6 and 255.6 mg/kg DM, respectively. In fact, soaking the seeds in 1% NaCl solution required 16 h more than in 1% citric acid to reach the same alkaloid content (about 1.0 g/kg DM [25]), leading to a greater leakage of the soluble phenolics.

The ANOVA showed a significant effect of the solvent on the total content of the conjugated phenolic compounds (the citric acid solution induced minor losses compared to the NaCl solution, probably for the same reason that has been described for the free fraction) but not on the individual compounds. On the other hand, the solvent effect on the insoluble-bound fraction was significant for the total content and all individual compounds, except for the naringenin derivatives; the samples that were treated with the citric acid solution lost more bound phenolics, except for the apigenin derivatives (present, however, in limited quantities) and, of course, naringenin.

### 3.2. Effect of Different Debittering Methods on Antioxidant Compounds

#### 3.2.1. Carotenoids and Tocopherols

Table 3 reports the carotenoid and tocopherol values in the Lot 2 seeds, which were debittered by the control methods with water or with NaCl solution, and the experimental method using different solvents and soaking times.

The samples showed a total carotenoid content of 16.37–25.08 mg/kg DM. The highest concentrations were detected in the lupins that were treated with 1% citric acid for 45 h (25.08 mg/kg DM). The most abundant compound was lutein, except for the samples that were treated with citric acid, where (α + β)-carotene was predominant. Contrary to the Lot 1 lupins, the seeds that were debittered with 1% NaCl presented a lower percentage of total carotenoids than those that were treated with 1% citric acid. Overall, long treatment times and, secondarily, high temperatures influenced the debittering results; the control method with salt induced the highest carotenoids loss, followed by the 57 h and 45 h 1% NaCl solutions, the control method with water and the 57 h and 45 h 1% citric acid solution.

Again, no tocotrienols were identified and the most abundant tocopherol was γ-tocopherol (over 98% of total tocopherols), with values in the range of 200.3–241.3 mg/kg DM, which was higher than those that were found in the debittered seeds of *L. albus* by Briceño Berru et al. [15] (131.0–218.4 mg/kg DM) and by Annicchiarico et al. [45] (107.0–153.0 mg/kg DM). The seeds that were treated with 1% NaCl for 45 h, or with the traditional method, showed significantly higher tocopherol content than the other samples. The control method with water limits the exposure to high temperatures to one hour, and this may have better preserved the tocopherols from thermal degradation; this would also explain the low content that was found in the seeds that were debittered with the control method with 0.5% NaCl, which involves 8 h of hydration at 80 °C and 1 h of cooking. In fact, this last approach led to concentrations that were lower than those of the seeds that were debittered with saline solutions but treated with 1 h of cooking and washed at 50 °C.

Among the samples that were treated with the experimental method, those that were debittered with 1% NaCl retained a significantly higher share of tocopherols compared to those that were debittered with 1% citric acid for the same amount of time. Within solvents, the seeds that were debittered for 45 h showed a higher concentration of tocopherols than those that were treated for 57 h. Hence, prolonging the soaking time increases the tocopherols loss.

#### 3.2.2. Phenolic Compounds

Table 4 reports the free, the conjugated and the bound phenolic compounds, and the total phenol content of the Lot 2 seeds that were debittered with the control methods (water or salt solution) and the experimental method. The total phenol content varied broadly (304.3–524.0 mg/kg DM) as a consequence of the very different treatment conditions. As already observed for the Lot 1 seeds, only genistein was identified with the corresponding retention time and spectrum, while the flavonoid derivatives that were detected were genistein, naringenin, diosmin, apigenin, and catechin, and the only phenolic acid derivative that was recorded was sinapic acid. Differently from the Lot 1 seeds, the genistein derivatives were absent among the free phenolics, and so were the apigenin derivatives in the soluble-conjugated phenolics, while genistein was observed in the bound phenolics. The total free phenolic content (106.9–303.4 mg/kg DM) was lower than the values (418.5–446.7 and 537.4 mg/kg DM) that were reported in non-debittered seeds by Siger et al. [55] and Kalogeropoulos et al. [56], respectively. The soluble-conjugated and insoluble-bound phenolic compounds were 74.2–118.7 mg/kg DM and 110.2–156.7 mg/kg DM, respectively.

**Figure 1:**
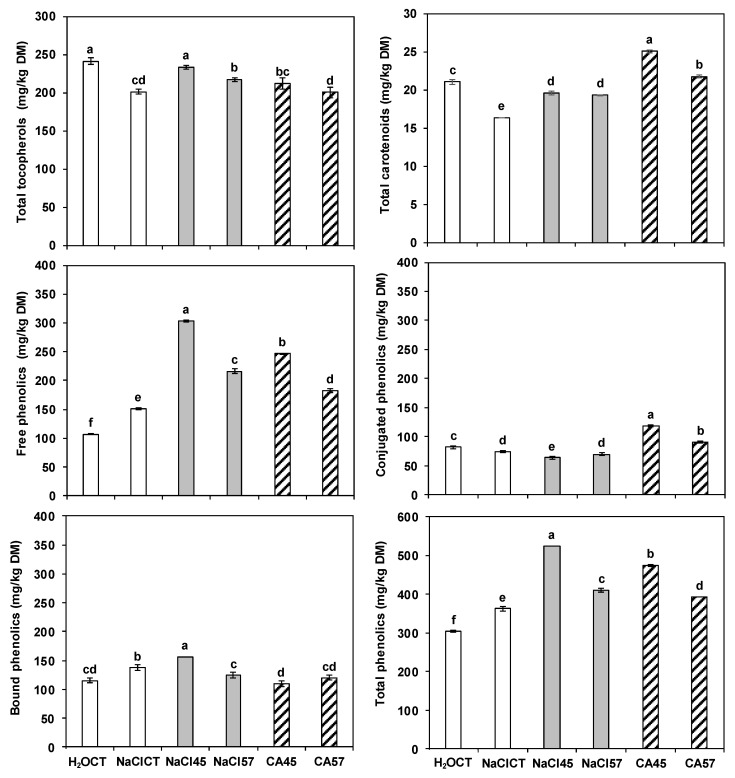
Total carotenoids, tocopherols, free phenolics, conjugated phenolics, bound phenolics, and overall phenolics of *Lupinus albus* seeds (Lot 2) debittered by the control methods with water (H_2_OCT; Erbaş [28]; Córdova-Ramos et al. [27]) or with NaCl solution (NaClCT; Villacrés et al. [31]), and by the debittering method proposed by Estivi et al. [25] using 1% NaCl solution (NaCl 45 and 57 h) or 1% citric acid solution (CA 45 and 57 h). The bars show the standard deviation, while the different letters indicate significant differences between samples according to the LSD test (*p* ≤ 0.05).

The LSD test revealed significant differences in the total polyphenol content among the debittering treatments (Figure 1). In particular, the treatments that better preserved the phenols were the shortest ones (45 h), with either 1% NaCl (524.0 mg/kg DM) or 1% citric acid (475.5 mg/kg DM). The control treatment with water induced the greatest phenolic loss (304.3 mg/kg DM), followed by the salt control method (362.9 mg/kg DM). The same ranking among treatments was recorded for the soluble-free fraction, which constituted the majority of the total phenolics and varied between 106.9 (traditional method) and 303.4 (1% NaCl solution for 45 h) mg/kg DM. Hence, the soaking time seems to be the main leaching cause of the soluble-free fraction.

Overall, even after debittering, the lupin seeds preserved a significant content of phenolic compounds, which are positively correlated to the antioxidant capacity [17,29]. The citric acid solutions better preserved the conjugated phenols (especially using the shortest soaking time), while the NaCl solutions behaved poorly. The contrary was instead evident for the bound phenols; all of the samples that were treated with salt solutions (either by the control method or by the experimental protocol) showed significantly higher levels of insoluble-bound polyphenols (124.4–156.7 mg/kg DM) compared to the samples that were treated with the traditional method (115.3 mg/kg DM) or with citric acid (110.2–120.0 mg/kg DM). The treatment with 1% NaCl for 45 h retained the greatest amount of bounds phenols.

## 4. Conclusions

The sonication decreased the content of carotenoids and soluble-free phenolics, but did not influence the tocopherols or the soluble-conjugated and insoluble-bound phenolic compounds. Nevertheless, the debittered lupins showed interesting quantities of tocopherols (172.8–241.3 mg/kg DM), carotenoids (10.9–25.1 mg/kg DM), and soluble-free (106.9–361.1 mg/kg DM), soluble-conjugated (93.9–118.9 mg/kg DM), and insoluble-bound (59.2–156.7 mg/kg DM) phenolic compounds. However, the soluble-free fraction was susceptible to increasing treatment times. The use of citric acid or sodium chloride optimized the process, leading to shortened treatment, reduced water consumption, and better preserved the antioxidant compounds in the debittered seeds.

Future investigations may take advantage of our results by assessing the bioaccessibility of the bioactive nutrients in commercial-like products from lupin flour. Furthermore, the experimental debittering method may be applied to germinated lupin seeds because the germination triggers the synthesis of antioxidants in legumes; however, conclusive evidence is still missing in lupins.

## Figures and Tables

**Table 1:** Carotenoid and tocopherol content (mg/kg DM; mean ± standard deviation) in *Lupinus albus* seeds (Lot 1) after debittering by the control method with water (Erbaş [28], modified by Córdova-Ramos et al. [27]) and by the method proposed by Estivi et al. [25], without and with ultrasound (US), and with different solvents (1% NaCl for 45 h or 1% citric acid for 28.5 h).

	Control H_2_O	1% NaCl	1% NaCl US	1% Citric Acid	1% Citric Acid US
		(0.98 ± 0.11 g alkaloids/kg DM)		(1.02 ± 0.07 g alkaloids/kg DM)	
*Carotenoids*					
(α + β)-carotene	2.25 ^ab^ ± 0.36	2.44 ^ab^ ± 0.09	2.41 ^ab^ ± 0.05	2.49 ^a^ ± 0.07	2.02 ^b^ ± 0.02
β-cryptoxanthin	0.18 ^c^ ± 0.02	0.11 ^d^ ± 0.00	0.10 ^d^ ± 0.00	0.42 ^b^ ± 0.00	0.50 ^a^ ± 0.03
Lutein	7.91 ^ab^ ± 0.86	8.74 ^a^ ± 0.47	8.35 ^a^ ± 0.03	8.05 ^a^ ± 0.27	6.83 ^b^ ± 0.03
Zeaxanthin	1.86 ^ab^ ± 0.14	1.95 ^a^ ± 0.10	1.94 ^ab^ ± 0.01	1.74 ^b^ ± 0.05	1.50 ^c^ ± 0.01
Total carotenoids	12.20 ^ab^ ± 1.38	13.23 ^a^ ± 0.66	12.80 ^a^ ± 0.01	12.70 ^a^ ± 0.39	10.85 ^b^ ± 0.08
*Tocopherols*					
α-tocopherol	0.45 ^b^ ± 0.10	0.46 ^ab^ ± 0.04	0.56 ^a^ ± 0.03	0.50 ^ab^ ± 0.01	0.44 ^b^ ± 0.00
β-tocopherol	1.36 ^b^ ± 0.51	1.01 ^b^ ± 0.17	0.85 ^b^ ± 0.25	2.64 ^a^ ± 0.02	2.09 ^a^ ± 0.15
γ-tocopherol	191.58 ± 27.96	188.73 ± 17.08	175.82 ± 0.06	182.93 ± 3.22	169.04 ± 3.38
δ-tocopherol	1.99 ± 0.40	1.77 ± 0.06	1.61 ± 0.12	1.56 ± 0.52	1.24 ± 0.08
Total tocopherols	195.38 ± 28.97	191.97 ± 16.92	178.83 ± 0.47	187.63 ± 3.75	172.82 ± 3.32

Different letters indicate significant differences between samples in the same row according to the LSD test (*p* ≤ 0.05).

**Table 2:** Phenolic content (mg/kg DM; mean ± standard deviation) in *Lupinus albus* seeds (Lot 1) after debittering by the control method with water (Erbaş [28], modified by Córdova-Ramos et al., [27]) and by the method proposed by Estivi et al. [25], without and with ultrasound (US), and with different solvents (1% NaCl for 45 h or 1% citric acid for 28.5 h).

	Control H_2_O	1% NaCl	1% NaCl US	1% Citric Acid	1% Citric Acid US
		(0.98 ± 0.11 g alkaloids/kg DM)		(1.02 ± 0.07 g alkaloids/kg DM)	
*Soluble-free phenolics*					
Genistein der.	20.88 ^e^ ± 0.46	66.12 ^c^ ± 0.03	54.33 ^d^ ± 1.21	103.21 ^b^ ± 0.27	117.92 ^a^ ± 1.94
Sinapic acid der.	120.60 ^a^ ± 0.18	107.49 ^a^ ± 0.48	89.30 ^b^ ± 1.57	85.91 ^b^ ± 7.16	66.40 ^c^ ± 12.07
Naringenin der.	nd	nd	nd	4.11 ^a^ ± 0.14	3.58 ^b^ ± 0.01
Diosmin der.	nd	nd	nd	3.94 ^a^ ± 0.06	3.24 ^b^ ± 0.00
Apigenin der.	30.53 ^e^ ± 0.08	101.90 ^c^ ± 1.08	92.08 ^d^ ± 1.97	163.96 ^a^ ± 3.38	134.99 ^b^ ± 0.88
Total free	172.02 ^e^ ± 0.21	275.51 ^c^ ± 1.59	235.70 ^d^ ± 1.61	361.13 ^a^ ± 4.25	326.12 ^b^ ± 11.00
*Soluble-conjugated phenolics*					
Genistein der.	33.63 ^c^ ± 2.58	82.80 ^b^ ± 10.78	97.76 ^ab^ ± 13.13	111.76 ^a^ ± 4.82	99.15 ^ab^ ± 1.71
Genistein	nd	nd	nd	1.55 ^a^ ± 0.06	0.44 ^b^ ± 0.05
Naringenin der.	5.46 ^a^ ± 0.39	1.97^b^ ± 0.73	1.83 ^b^ ± 0.45	2.08 ^b^ ± 0.20	1.25 ^b^ ± 0.03
Catechin der.	0.41 ^c^ ± 0.04	nd	nd	3.54 ^a^ ± 0.11	2.53 ^b^ ± 0.05
Apigenin der.	39.49 ^a^ ± 3.02	0.52 ^b^ ± 0.10	0.54 ^b^ ± 0.02	nd	nd
Total conjugated	78.99 ^c^ ± 6.03	85.28 ^bc^ ± 11.6	100.13 ^abc^ ± 12.7	118.94^a^ ± 5.06	103.37 ^ab^ ± 1.68
*Insoluble-bound phenolics*					
Genistein der.	40.41 ^b^ ± 6.07	53.71 ^a^ ± 4.37	52.50 ^a^ ± 3.45	29.23 ^b^ ± 4.23	34.76 ^b^ ± 3.64
Naringenin der.	14.27 ± 0.57	18.39 ± 3.39	19.02 ± 1.15	15.25 ± 1.69	14.63 ± 1.04
Catechin der.	26.24 ^a^ ± 0.53	28.25 ^a^ ± 0.98	26.5 ^a^ ± 2.06	8.74 ^b^ ± 0.95	8.38 ^b^ ± 0.97
Apigenin der.	1.77 ^b^ ± 0.13	0.84 ^b^ ± 0.22	0.90 ^b^ ± 0.09	6.01 ^a^ ± 0.82	5.91 ^a^ ± 0.12
Total bound	82.69 ^b^ ± 7.30	101.19 ^a^ ± 8.53	98.92 ^ab^ ± 6.57	59.23 ^c^ ± 7.69	63.68 ^c^ ± 3.83
*Total phenolics*	333.69 ^d^ ± 13.13	461.98 ^bc^ ± 4.66	434.75 ^c^ ± 17.66	539.29 ^a^ ± 6.88	493.17 ^b^ ± 16.51

nd, below the detection limit. The different letters indicate significant differences between samples in the same row according to the LSD test (*p* ≤ 0.05).

**Table 3:** Tocopherol and carotenoid content (mg/kg DM; mean ± standard deviation) in *Lupinus albus* seeds (Lot 2) debittered by the control methods with water (Erbaş [28], modified by Córdova-Ramos et al. [27]) or with 0.5% NaCl [31] and the experimental method with different solvents [25].

	Control H_2_O	Control 0.5% NaCl	1% NaCl 45 h	1% NaCl 57 h	1% Citric Acid 45 h	1% Citric Acid 57 h
*Carotenoids*						
(α + β)-carotene	7.86 ^c^ ± 0.23	5.30 ^e^ ± 0.22	7.10 ^d^ ± 0.04	6.89 ^d^ ± 0.03	12.01 ^a^ ± 0.15	9.63 ^b^ ± 0.06
β-cryptoxanthin	1.58 ^c^ ± 0.04	1.12 ^d^ ± 0.19	1.29 ^d^ ± 0.01	1.21 ^d^ ± 0.03	3.05 ^a^ ± 0.05	2.60 ^b^ ± 0.06
Lutein	9.33 ^a^ ± 0.09	8.06 ^b^ ± 0.04	9.17 ^a^ ± 0.19	9.35 ^a^ ± 0.06	7.97 ^b^ ± 0.02	7.70 ^c^ ± 0.05
Zeaxanthin	2.30 ^a^ ± 0.06	1.90 ^c^ ± 0.04	2.07 ^b^ ± 0.03	1.89 ^c^ ± 0.01	2.06 ^b^ ± 0.03	1.85 ^c^ ± 0.03
*Tocopherols*						
α-tocopherol	0.26 ^b^ ± 0.08	0.47 ^a^ ± 0.03	0.51^a^ ± 0.01	0.50 ^a^ ± 0.00	0.29 ^b^ ± 0.02	0.31 ^b^ ± 0.02
β-tocopherol	1.13 ^a^^b^ ± 0.10	1.30 ^a^ ± 0.16	0.56^d^ ± 0.00	0.52 ^d^ ± 0.01	0.99 ^b^^c^ ± 0.02	0.99 ^c^ ± 0.07
γ-tocopherol	237.77 ^a^ ± 4.34	197.33 ^c^^d^ ± 3.07	230.53^a^ ± 2.66	214.27 ^b^ ± 2.65	208.35 ^b^^c^ ± 6.91	196.79 ^d^ ± 6.33
δ-tocopherol	2.09 ^b^^c^ ± 0.02	1.98 ^c^^d^ ± 0.01	1.82^d^ ± 0.08	2.26 ^b^ ± 0.03	2.45 ^a^ ± 0.09	2.23 ^b^ ± 0.15

Different letters indicate significant differences between samples in the same row according to the LSD test (*p* ≤ 0.05).

**Table 4:** Phenolic content (mg/kg DM; mean ± standard deviation) in *Lupinus albus* seeds (Lot 2) debittered by the control methods with water (Erbaş [28], modified by Córdova-Ramos et al. [27]) or with 0.5% NaCl [31], and the experimental method with different solvents [25].

	Control H_2_O	Control 0.5% NaCl	1% NaCl 45 h	1% NaCl 57 h	1% Citric Acid 45 h	1% Citric Acid 57 h
*Soluble-free phenolics*						
Sinapic acid der.	53.14 ^c^ ± 0.33	50.43 ^c^ ± 0.48	134.15 ^a^ ± 4.20	82.60 ^b^ ± 1.86	32.10 ^d^ ± 1.56	12.76 ^e^ ± 0.34
Naringenin der.	2.58 ^a^ ± 0.04	2.27 ^b^ ± 0.03	2.37 ^b^ ± 0.01	2.27 ^b^ ± 0.03	1.73 ^c^ ± 0.06	1.16 ^d^ ± 0.05
Diosmin der.	1.31 ^f^ ± 0.04	1.63 ^e^ ± 0.04	3.62 ^b^ ± 0.05	2.76 ^c^ ± 0.05	4.81 ^a^ ± 0.07	2.43 ^d^ ± 0.05
Apigenin der.	49.83 ^e^ ± 1.41	96.66 ^d^ ± 1.67	163.25 ^b^ ± 2.72	128.73 ^c^ ± 1.68	207.93 ^a^ ± 2.26	165.97 ^b^ ± 2.79
*Soluble-conjugated phenolics*						
Genistein der.	73.04 ^c^ ± 2.59	65.63 ^d^ ± 1.14	57.01 ^e^ ± 2.01	63.22 ^d^ ± 2.86	105.62 ^a^ ± 1.60	81.18 ^b^ ± 1.60
Genistein	2.68 ^e^ ± 0.02	3.71 ^c^ ± 0.02	2.56 ^f^ ± 0.03	3.15 ^d^ ± 0.03	4.17 ^a^ ± 0.02	3.97 ^b^ ± 0.03
Naringenin der.	6.36 ^a^ ± 0.09	2.68 ^e^ ± 0.05	2.80 ^d^^e^ ± 0.13	3.01 ^d^ ± 0.19	4.87 ^b^ ± 0.11	3.61 ^c^ ± 0.06
Catechin der.	nd	2.22 ^b^ ± 0.06	1.54 ^d^ ± 0.03	0.88 ^e^ ± 0.03	4.06 ^a^ ± 0.05	1.98 ^c^ ± 0.07
*Insoluble-bound phenolics*						
Genistein der.	78.23 ^c^ ± 2.76	94.50 ^b^ ± 2.87	112.91 ^a^ ± 1.43	83.44 ^c^ ± 5.57	83.91 ^c^ ± 4.85	93.91 ^b^ ± 4.19
Genistein	2.13 ^a^ ± 0.02	1.06 ^d^ ± 0.03	1.51 ^c^ ± 0.09	1.75 ^b^ ± 0.09	1.77 ^b^ ± 0.04	1.52 ^c^ ± 0.01
Naringenin der.	10.89 ^c^ ± 0.91	13.24 ^a^^b^ ± 1.01	14.42 ^a^ ± 1.48	11.42 ^b^^c^ ± 0.84	11.44 ^b^^c^ ± 0.22	11.66 ^b^^c^ ± 0.15
Catechin der.	23.94 ^b^ ± 0.91	28.57 ^a^ ± 0.97	27.39 ^a^ ± 0.13	27.37 ^a^ ± 0.05	11.70 ^c^ ± 0.06	11.63 ^c^ ± 0.06
Apigenin der.	0.15 ^d^ ± 0.04	0.30 ^c^ ± 0.03	0.49 ^b^ ± 0.06	0.42 ^b^ ± 0.01	1.35 ^a^ ± 0.00	1.27 ^a^ ± 0.01

nd, below the detection limit. The different letters indicate significant differences between samples in the same row according to the LSD test (*p* ≤ 0.05).

## Data Availability

The data presented in this study are available in the article and supplementary materials.

## Supplementary Materials

The following supporting information can be downloaded at: https://www.mdpi.com/article/10.3390/antiox11122481/s1, Table S1: Analysis of variance (mean square) and LSD test of tocopherol and carotenoid content (mg/kg DM; mean ± standard error) of *Lupinus albus* seeds (Lot 1) after debittering both with and without ultrasound (US), and with different solvents.; Table S2: Analysis of variance (mean square) and LSD test of the polyphenol content (mg/kg DM; mean ± standard error) of *Lupinus albus* seeds (Lot 1) after debittering both with and without ultrasound (US) and with different solvents.
