# Supplementary material for: Effect of Debittering with Different Solvents and Ultrasound on Carotenoids, Tocopherols, and Phenolics of Lupinus albus Seeds
Source: Antioxidants (Basel). 2022 Dec 16;11(12):2481. doi: 10.3390/antiox11122481 (PMC9774723; doi:10.3390/antiox11122481)
Supplement: Supplementary file 1 [file antioxidants-11-02481-s001.zip › antioxidants-2095235-SI.pdf]

Supplementary Table S1. Analysis of variance (mean square) and LSD test of tocopherols and carotenoids content (mg/kg DM; mean ± standard error) of *Lupinus albus* seeds (Lot 1) after debittering without and with ultrasound (US), and with different solvents.

	ANOVA				LSD Treatment		LSD Solvent	
	Treatment (T)	Solvent (S)	T x S	Error	Without US	With US	1% NaCl	1% Citric acid
d.f.	1	1	1	4				
(α + β)-carotene	0.13 **	0.054 *	0.097 **	0.0040	2.46 ^{a} ± 0.04	2.21 ^{b} ± 0.11	2.42 ^{a} ± 0.03	2.26 ^{b} ± 0.14
β-cryptoxanthin	0.0028 *	0.26 ***	0.0036 *	0.00021	0.26 ^{b} ± 0.09	0.30 ^{a} ± 0.12	0.11 ^{b} ± 0.00	0.46 ^{a} ± 0.02
Lutein	1.30 *	2.4 **	0.34	0.073	8.39 ^{a} ± 0.25	7.59 ^{b} ± 0.44	8.54 ^{a} ± 0.18	7.44 ^{b} ± 0.36
Zeaxanthin	0.030 *	0.21 **	0.025 *	0.0032	1.84 ^{a} ± 0.07	1.72 ^{b} ± 0.13	1.95 ^{a} ± 0.03	1.62 ^{b} ± 0.07
Total carotenoids	2.6 *	3.1 *	0.99	0.15	12.97 ^{a} ± 0.27	11.83 ^{b} ± 0.56	13.02 ^{a} ± 0.23	11.77 ^{b} ± 0.55
α-tocopherol	0.00080	0.0025	0.011*	0.00083	0.48 ± 0.02	0.50 ± 0.03	0.51 ± 0.03	0.47 ± 0.02
β-tocopherol	0.25 *	4.2 **	0.080	0.023	1.83 ^{a} ± 0.47	1.47 ^{b} ± 0.37	0.93 ^{b} ± 0.10	2.36 ^{a} ± 0.17
γ-tocopherol	359	79	0.50	78	185.83 ± 5.29	172.43 ± 2.19	182.28 ± 6.18	175.99 ± 4.23
δ-tocopherol	0.10	0.15	0.011	0.069	1.67 ± 0.16	1.42 ± 0.11	1.69 ± 0.06	1.40 ± 0.18
Total tocopherols	389	53	1.4	78	189.80 ± 5.16	175.82 ± 1.99	185.40 ± 6.19	180.22 ± 4.51

d.f.: degrees of freedom; *, $p \leq 0.05$; **, $p \leq 0.01$; ***, $p \leq 0.001$. Different letters indicate significant differences between samples according to the LSD test ($p \leq 0.05$).

Supplementary Table S2. Analysis of variance (mean square) and LSD test of the polyphenols content (mg/kg DM; mean ± standard error) of *Lupinus albus* seeds (Lot 1) after debittering without and with ultrasound (US), and with solvents different.

	ANOVA				LSD Treatment		LSD solvent	
	Treatment (T)	Solvent (S)	T x S	Error	Without US	With US	1% NaCl	1% Citric acid
d.f.	1	1	1	4				
Free phenolics								
Genistein der.	4.2	5068 ***	351 ***	1.3	84.67 ± 10.71	86.12 ± 18.37	60.22 [b] ± 3.42	110.56 [a] ± 4.28
Sinapic acid der.	711 *	989 *	0.86	50	96.70 [a] ± 6.57	77.85 [b] ± 7.49	98.39 [a] ± 5.27	76.15 [b] ± 6.94
Naringenin der.					4.11 [a] ± 0.07	3.58 [b] ± 0.00	nd	3.85 ± 0.16
Diosmin der.					3.94 [a] ± 0.03	3.24 [b] ± 0.00	nd	3.59 ± 0.20
Apigenin der.	753 ***	5511 ***	183 **	4.3	132.93 [a] ± 17.95	113.53 [b] ± 12.40	96.99 [b] ± 2.91	149.47 [a] ± 8.43
Total free	2798 ***	15495 ***	12	36	318.32 [a] ± 24.75	280.91 [b] ± 26.30	255.60 [b] ± 11.51	343.62 [a] ± 10.66
Conjugated phenolics								
Genistein der.	2.8	461	380	79	97.28 ± 9.03	98.46 ± 3.84	90.28 ± 6.53	105.46 ± 3.93
Genistein					1.55 [a] ± 0.03	0.44 [b] ± 0.03	nd	1.00 ± 0.32
Naringenin der.	0.46	0.11	0.24	0.19	2.02 ± 0.22	1.54 ± 0.21	1.90 ± 0.25	1.67 ± 0.25
Catechin der.					3.54 [a] ± 0.05	2.53 [b] ± 0.03	nd	3.04 ± 0.30
Apigenin der.					0.52 ± 0.05	0.54 ± 0.01	0.53 ± 0.03	nd
Total conjugated	0.26	681*	463	81	102.11 ± 10.38	101.75 ± 3.81	92.71 [b] ± 6.56	111.16 [a] ± 4.75
Bound phenolics								
Genistein der.	9.4	891 **	23	16	41.47 ± 7.28	43.63 ± 5.32	53.10 [a] ± 1.64	31.99 [b] ± 2.27
Naringenin der.	0.000013	28	0.79	4.2	16.82 ± 1.42	16.82 ± 1.34	18.71 ± 1.05	14.94 ± 0.60
Catechin der.	2.2	708 ***	0.96	1.8	18.49 ± 5.65	17.44 ± 5.27	27.37 [a] ± 0.83	8.56 [b] ± 0.41
Apigenin der.	0.00080	52 ***	0.011	0.19	3.43 ± 1.51	3.41 ± 1.45	0.87 [b] ± 0.07	5.96 [a] ± 0.24
Total bound	2.4	2980 **	23	47	80.21 ± 12.56	81.30 ± 10.41	100.05 [a] ± 3.18	61.45 [b] ± 2.79
Total phenolics	2690*	9212 **	179	163	500.64 [a] ± 22.45	463.96 [b] ± 18.25	448.37 [b] ± 9.46	516.23 [a] ± 14.28

d.f.: degrees of freedom; der.: derivative; nd: below the detection limit. *, $p \leq 0.05$; **, $p \leq 0.01$; ***, $p \leq 0.001$. Different letters indicate significant differences between samples according to the LSD test ($p \leq 0.05$).
